# Changes in Viral Dynamics Following the Legal Relaxation of COVID‐19 Mitigation Measures in Japan From Children to Adults: A Single Center Study, 2020–2023

**DOI:** 10.1111/irv.13278

**Published:** 2024-03-21

**Authors:** Yosuke Hirotsu, Yuki Nagakubo, Makoto Maejima, Masahiro Shibusawa, Kazuhiro Hosaka, Hitomi Sueki, Hitoshi Mochizuki, Masao Omata

**Affiliations:** ^1^ Genome Analysis Center Yamanashi Central Hospital Kofu Yamanashi Japan; ^2^ Division of Microbiology in Clinical Laboratory Yamanashi Central Hospital Kofu Yamanashi Japan; ^3^ Division of Genetics and Clinical Laboratory Yamanashi Central Hospital Kofu Yamanashi Japan; ^4^ Central Clinical Laboratory Yamanashi Central Hospital Kofu Yamanashi Japan; ^5^ Department of Gastroenterology Yamanashi Central Hospital Kofu Yamanashi Japan; ^6^ The University of Tokyo Tokyo Japan

**Keywords:** COVID‐19, enterovirus, influenza, rhinovirus, RSV

## Abstract

**Introduction:**

Respiratory infections are an ongoing global health challenge. The COVID‐19 pandemic triggered global nonpharmacological measures that reshaped public health. In Japan, the shift from legal to individual discretion in pandemic management started on May 8, 2023. However, it still unknown how the relaxation of measures affects respiratory pathogens across age groups.

**Methods:**

We collected 16,946 samples from 13,526 patients between February 2020 and September 2023, analyzing the circulating respiratory pathogen dynamics using FilmArray respiratory panel.

**Results:**

Our analysis revealed significant increases in the positivity rates of respiratory pathogens across multiple age groups after relaxation. The pathogens including adenovirus, *Bordetella pertussis* , parainfluenza 2 and parainfluenza 4 showed increased positivity predominantly in children aged under 10 years. Conversely, some pathogens including human metapneumovirus, rhinovirus/enterovirus, and respiratory virus (RSV) increased in broad range of age groups. SARS‐CoV‐2 positivity rates decreased in children under 10 years but increased in those aged over 60 years.

**Discussion:**

Age‐stratified analysis reveals a dynamic pattern of circulating pathogen in each age group after relaxation measures. This study provides essential epidemiologic data that can guide strategies to protect different age groups and effectively respond to respiratory infections in post‐COVID‐19 era.

## 1 Introduction

Respiratory infections are a persistent global health concern, with their patterns and prevalence changing dynamically over time in response to environmental, behavioral, and healthcare factors. The global COVID‐19 pandemic led nations worldwide to implement a variety of nonpharmacological measures to mitigate viral infections, reshaping the landscape of public health responses [1, 2, 3, 4]. During these periods of infection control measures, the epidemiology of infectious diseases underwent significant changes [5, 6, 7, 8].

In Japan, people who tested positive for SARS‐CoV‐2 and their close contacts were required by the Infectious Disease Control Law to refrain from leaving their homes after the COVID‐19 pandemic [9, 10]. However, a significant change in the legal framework for infectious diseases occurred on May 8, 2023, marking a notable shift in the country's approach to pandemic management. Specifically, while the Japanese government had previously mandated legally based self‐restrictive measures, it moved away from a unified response strategy for COVID‐19 infection control. Instead, the focus shifted to individual and businesses discretion.

As a result of these policy shifts, there is a growing interest in understanding how the dynamics of respiratory viruses have changed [11]. Preliminary evidences suggest that there has been an increase in respiratory‐related viruses after the relaxation of strict containment measures [12, 13]. This relaxation of COVID‐19 measures represents a critical juncture in the country's approach to pandemic management and public health measures. Understanding these changes and the dynamics of circulating respiratory pathogens is critical because it provides insight into the evolving post‐pandemic landscape and the adaptation of public health strategies.

To this end, we present a comprehensive analysis of the positivity rates of respiratory pathogens and examine the impact of these regulatory relaxations on infection interventions in different age groups from infants to the elderly. These findings provide essential data to inform public health policies and interventions critical to protecting different age groups and guiding effective responses to respiratory infections in the post‐COVID‐19 era.

## 2 Methods

### 2.1 Patients and Samples

From February 2020 and September 30, 2023, we collected 16,946 samples from 13,526 patients who visited Yamanashi Central Hospital, Japan. Of 16,946 samples, 15,706 samples were collected during the pre‐relaxation period (from February 19, 2020, to May 8, 2023), and 1240 samples were collected during the post‐relaxation period (from May 8, 2023, to September 30, 2023) (Table S1). The median age of participants was 59 years (IQR: 26–79), with 9190 men (54%) and 7756 women (46%). Most of these patients showed at least one symptom of fever, headache, fatigue, nasal congestion, nasal discharge, sneezing, sore throat, and/or cough. Some asymptomatic individuals who had close contact with an individual infected with SARS‐CoV‐2 were also included. The necessity of testing was determined by physicians, and all anonymized data from testing were subject to analysis. More than two samples were taken from the same patient at different time points in some cases. The data were collected from the electronic records after testing. Some of the historical data contain samples that overlap with the data we previously reported [14].

The Institutional Review Board of Yamanashi Central Hospital approved this study, which complied with the Declaration of Helsinki and used the opt‐out consent method with written notice for all patients (approval number: C2019‐30 and C29‐19). This study used data obtained in the regular course of medical diagnosis, and no additional procedures were required of the patients during the study. To avoid identifying personal information, the results were obtained and analyzed from the data of a large number of people and do not include personal data. The requirement for written informed consent was waived because this was an observational study.

### 2.2 FilmArray Respiratory Panel (RP)

All nasopharyngeal swab samples were collected using cotton swabs and were stored in viral transport medium (Copan, Murrieta, CA, USA) or the ALLTM set medium (SG Medical, Seoul, Republic of Korea). We performed a multiplex PCR assay targeting 18 viruses and 3 bacteria species using FilmArray RP v1.7 (bioMérieux, Marcy‐l'Etoile, France). The newer version of FilmArray RP v.2.1 detected SARS‐CoV‐2 along with other pathogens detectable by the previous version (v.1.7). In brief, buffer and 300 μL of medium were injected into the FilmArray pouch. The reaction proceeded on the FilmArray Torch system automatically. If the internal positive control was not detected (failed or invalid), we repeated the test using the same viral transport medium. FilmArray RP does not distinguish between rhinoviruses and enteroviruses. For this study, when a rhinovirus or enterovirus was detected, they were treated as a single pathogen for convenience. As SARS‐CoV‐2 could not be detected by previous version (v.1.7), additional SARS‐CoV‐2 PCR test was conducted (see below).

### 2.3 Viral Nucleic Acid Extraction

We automatically isolated total nucleic acid using the same viral transport medium used in the FilmArray RP assay. This isolation was conducted with the MagMax Viral/Pathogen Nucleic Acid Isolation Kit (Thermo Fisher Scientific, Waltham, MA, USA) on a KingFisher Duo Prime system (Thermo Fisher Scientific). Briefly, we added 200 μL of viral transport medium, 5 μL of proteinase K, 265 μL of binding solution, 10 μL of total nucleic acid‐binding beads, 0.5 mL of wash buffer, and 0.5–1 mL of 80% ethanol to each well of a deep‐well 96‐well plate. The nucleic acids were eluted with 70 μL of elution buffer. The total nucleic acids were immediately subjected to quantitative reverse transcription PCR (RT‐qPCR).

### 2.4 RT‐qPCR

To detect SARS‐CoV‐2, we performed one‐step RT‐qPCR in accordance with the protocol developed by the National Institute of Infectious Diseases in Japan. This PCR method amplifies the nucleocapsid gene of SARS‐CoV‐2 (NC_045512.2). The reaction mixture was composed of 5 μL of 4× TaqMan Fast Virus 1‐Step Master Mix (Thermo Fisher Scientific), 1.0 μL of 10 μM forward primer (5′‐AAATTTTGGGGACCAGGAAC‐3′), 1.4 μL of 10 μM reverse primer (5′‐TGGCAGCTGTGTAGGTCAAC‐3′), 0.8 μL of 5 μM probe (5′‐FAM‐ATGTCGCGCATTGGCATGGA‐TAMRA‐3′), 6.8 μL of nuclease‐free water, and 5 μL of nucleic acid sample in a 20‐μL total volume [15, 16]. The human ribonuclease P protein subunit p30 (RPP30) gene was used as an internal positive control (Integrated DNA Technologies, Coralville, IA, USA).

The RT‐qPCR assays were conducted on a StepOnePlus Real‐Time PCR system (Thermo Fisher Scientific) with the following cycle conditions: 50°C for 5 min for reverse transcription, 95°C for 20 s, and 45 cycles of 95°C for 3 s and 60°C for 30 s. The threshold was set at 0.2. In accordance with the national protocol (version 2.9.1), samples were assessed as positive if a visible amplification plot was observed and assessed as negative if no amplification was observed.

### 2.5 Statistics

All statistical tests and visualizations were performed with R, version 4.1.1 (R Foundation for Statistical Computing) or RStudio (https://www.rstudio.com/). The following R packages were used for data cleaning, analysis, and visualization: ggplot2 (v3.3.5), dplyr (v1.0.7), tidyr (v1.1.3), patchwork (v1.1.1), gtsummary (v1.5.2), ggtext (v.0.1.2), ggrepel (v.0.9.3), ggstatsplot (v.0.12.0), incidence2 (v.2.2.1), fmsb (v.0.7.5), and cowplot (v.1.1.1). The 95% confidence intervals (CIs) were calculated, and *p*‐values <0.05 were considered to be statistically significant. Fisher's exact test with cross‐tabulation was performed to determine the pathogen infection status by age group. To analyze the changes in positivity rates before and after relaxation, we divided the data into prerelaxation and postrelaxation sets. Subsequently, we conducted predictions by fitting a linear regression model for each age group.

## 3 Results

### 3.1 Changes in Positivity Rates by Age Before and After Relaxation

**FIGURE 1:**
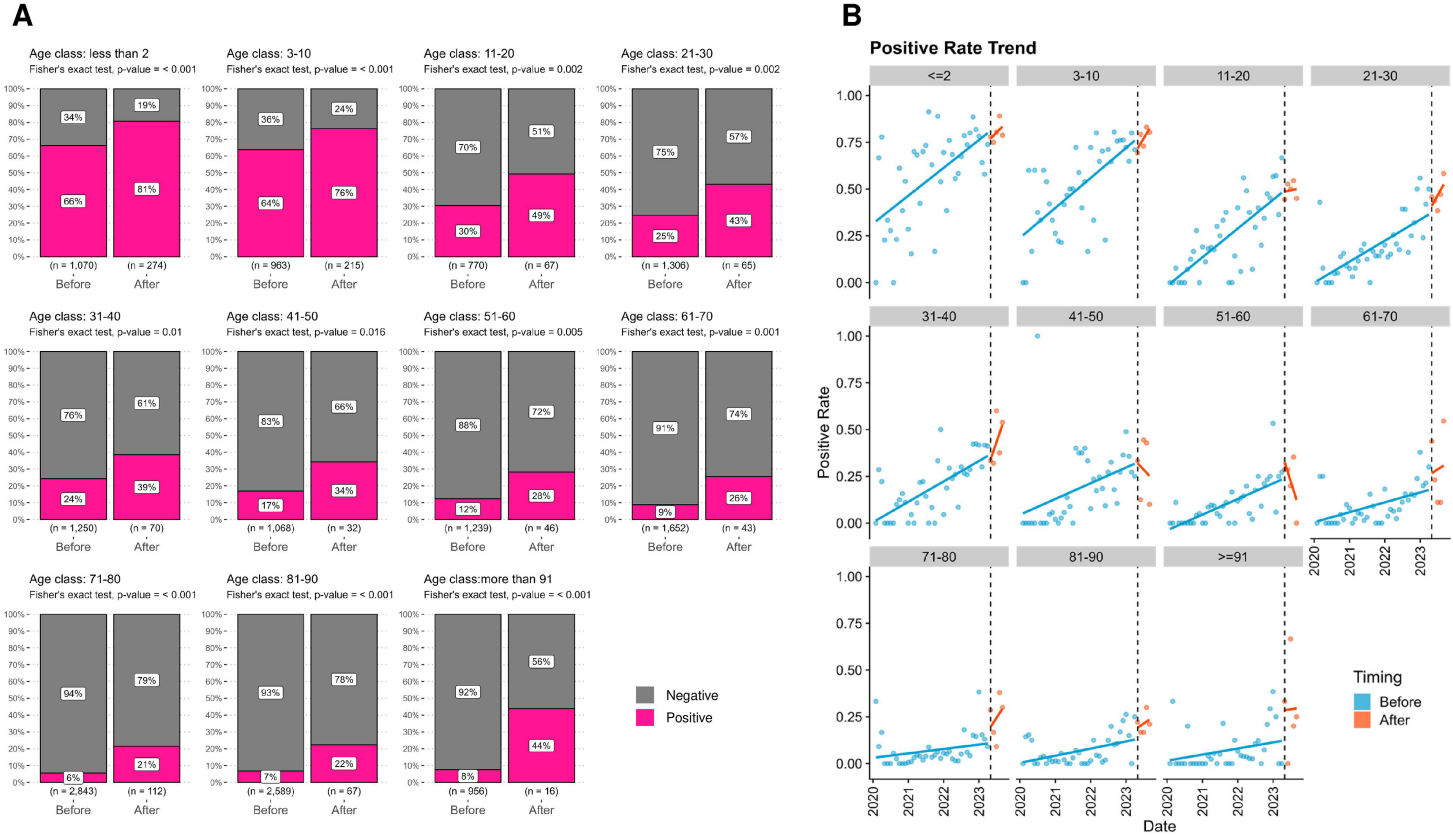
Changes in age‐stratified positivity rates before and after regulatory relaxation. (A) The bar chart illustrates the changes in positivity rates before and after relaxation. The values within the bars represent positivity rates (in pink) and negativity rates (in gray), with the sample size indicated below each graph. *p*‐values were calculated using chi‐squared tests. (B) Temporal changes in monthly positivity rates before and after relaxation. Each dot represents the positivity rate for each month, while the lines depict predictions based on a linear regression model. Blue dots indicate data before relaxation, orange dots represent data after relaxation. The black dashed line indicates the point of relaxation in May 2023.

To assess changes in the positivity rates of the pathogens detected within these samples, we performed an age‐stratified evaluation. The results showed the following changes in positivity rates before and after relaxation. Age under 2 years: 66% to 80% (chi‐squared test, *p* < 0.001); 3–10 years: 64% to 77% (*p* < 0.001); 11–20 years: 30% to 48% (*p* = 0.001); 21–30 years: 25% to 45% (*p* < 0.001); 31–40 years: 24% to 41% (*p* = 0.002); 41–50 years: 17% to 29% (*p* = 0. 06); 51–60 years: 12% to 27% (*p* = 0.007); 61–70 years: 9% to 31% (*p* < 0.001); 71–80 years: 6% to 23% (*p* < 0.001); 81–90 years: 7% to 22% (*p* < 0.001); over 91 years: 8% to 40% (*p* < 0.001) (Figure 1A). With the exception of the 41–50 and 51–60 age groups, an upward trend in positivity rates was observed in all age groups after relaxation compared with before relaxation (Figure 1B). The younger population had a higher increase in positivity rates. These results indicate that the positivity rates of respiratory pathogens have increased in multiple age groups after the regulatory relaxation of measures, and this upward trend continues even after the relaxation.

### 3.2 Changes in Age‐Specific Positivity Rates Before and After Relaxation

**FIGURE 2:**
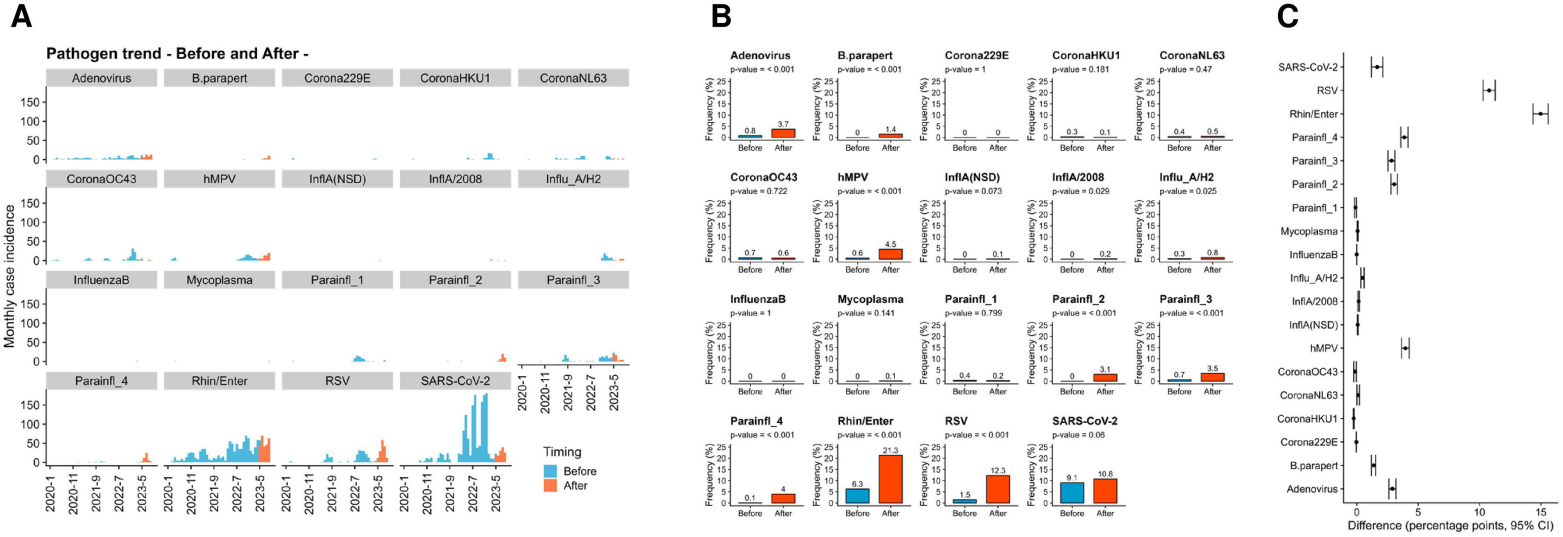
Changes in respiratory pathogens before and after regulatory relaxation. (A) Bar graphs show the monthly trends in the number of infections for each pathogen. Blue represents data before relaxation, and orange represents data after relaxation. (B) Bar graphs illustrate the changes in detection frequency (%) before and after relaxation. The values above the bars represent the frequency percentages. (C) The bar graph displays the percentage point difference between after and before relaxation. Error bars indicate 95% confidence intervals (CI).

To examine changes in the pathogens detected before and after the relaxation period, we aggregated the number of positive samples throughout the observation period. Before the relaxation, SARS‐CoV‐2 was detected more frequently, but its overall positivity rates decreased after the relaxation (Figure 2A). In contrast, adenovirus, *Bordetella pertussis* , human metapneumovirus, parainfluenza 2, parainfluenza 3, parainfluenza 4, rhinovirus/enterovirus, and RSV had significantly increased (*p* < 0.001, chi‐squared test) (Figure 2A,B). The percentage point differences for each pathogen before and after relaxation were adenovirus (2.9, 95% CI: 2.6–3.2), *Bordetella pertussis* (1.4, 95% CI: 1.2–1.5), human metapneumovirus (4.0, 95% CI: 3.6–4.3), parainfluenza 2 (3.1, 95% CI: 2.8–3.3), parainfluenza 3 (2.8, 95% CI: 2.6–3.1), parainfluenza 4 (3.9, 95% CI: 3.6–4.2), rhinovirus/enterovirus (15.0, 95% CI: 14.4–15.6), and RSV (10.8, 95% CI: 10.3–11.3) (Figure 2C).

### 3.3 Prominent Pathogen Changes in Pediatrics

**FIGURE 3:**
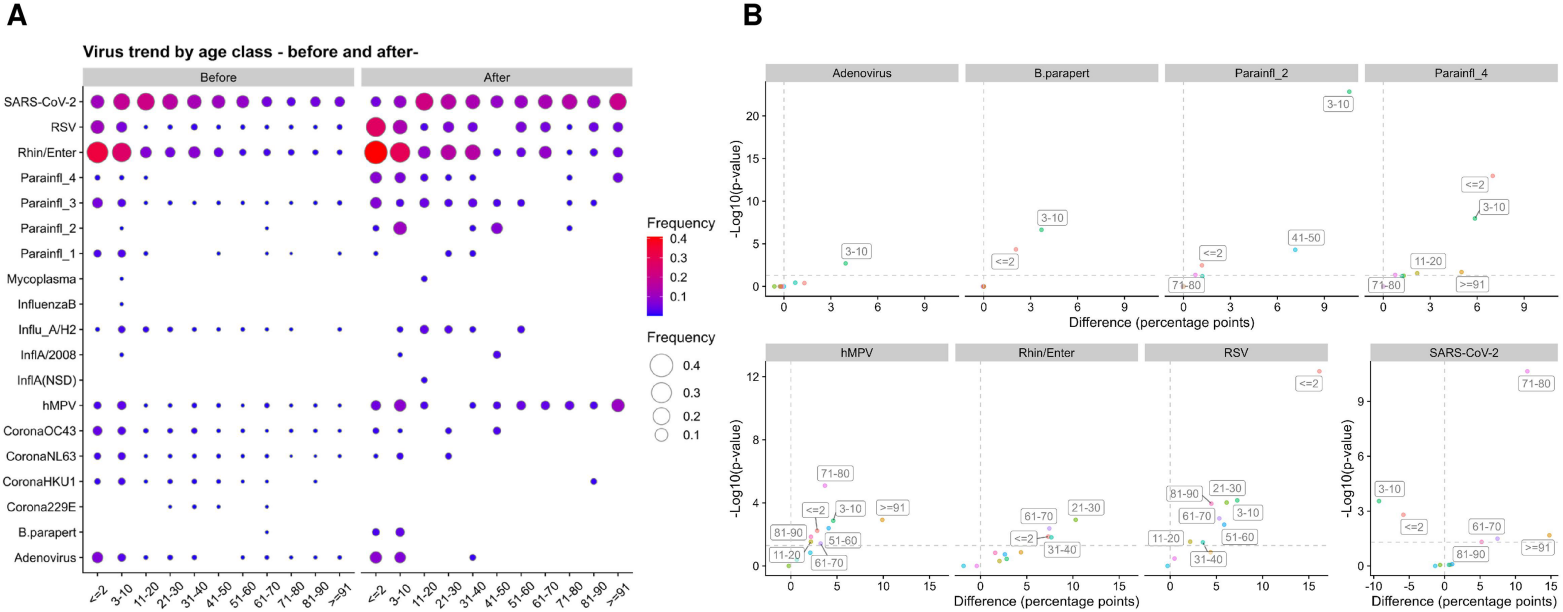
Changes in respiratory pathogen infections before and after regulatory relaxation in different age groups. (A) Balloon plots show age‐specific positivity rates before and after relaxation for each respiratory infection. The size and color intensity of the circles represent the frequency. (B) Scatter plots show the relationship between changes before and after relaxation and their statistical significance. The horizontal axis represents the difference in frequencies (difference) between after and before relaxation in percentage points. The vertical axis shows the reciprocal of the log_10_ value of the p‐value calculated by the chi‐squared test, indicated as −log_10_(*p*‐value).

An age‐stratified analysis of pathogen positivity rates before and after relaxation was performed for pediatrics patients (under 10 years) (Figure 3A,B and Table S2). Of note, the pediatric population had increased positivity rate after relaxation for adenovirus, *Bordetella pertussis* , parainfluenza 2, and parainfluenza 4. The changes in positivity rates before and after relaxation were as follows: Adenovirus increased from 2.7% (95% CI: 1.9%–3.9%) to 6.6% (4.2%–10%) (*p* = 0.002, chi‐squared test) in 3–10 years; *Bordetella pertussis* increased from 0% (0%–0.36%) to 2.1% (1.0%–4.2%) under 2 years (*p* < 0.001), and from 0% (0%–0.40%) to 3.7% (2.0%–6.7%) in 3–10 years (*p* < 0.001); parainfluenza 2 increased from 0% (0%–0.36%) to 1.2% (0.46%–3.0%) under 2 years (*p* = 0.003) and from 0.1% (0.018%–0.59%) to 11% (7.6%–15%) in 3–10 years (*p* < 0.001); parainfluenza 4 increased from 0.65% (0.32%–1.3%) to 7.7% (5.3%–11%) under 2 years (*p* < 0.001), and from 0.42% (0.16%–1.1%) to 6.3% (4.0%–9.8%) in 3–10 years (*p* < 0.001). Conversely, the positivity rates of coronavirus HKU1, coronavirus OC43, and parainfluenza 1 were decreased after relaxation (Figure S1).

### 3.4 Prominent Pathogen Changes Across a Wide Age Range

We next assessed the pathogens that exhibited an increase in positivity rates across various age groups after relaxation (Figure 3A,B, Figure S2, and Table S2). The data showed hMPV, rhinovirus/enterovirus, and RSV were increased. hMPV increased from 2.2% (95% CI: 1.4%–3.2%) to 5.0% (3.1%–7.9%) under 2 years (chi‐squared test, *p* = 0.006); 3.5% (2.5%–4.9%) to 8.1% (5.4%–12%) in 3–10 years (*p* = 0.001); 0.1% (0%–0.7%) to 2.3% (0.6%–8%) in 11–20 years (*p* = 0.029); 0.08% (0%–0.5%) to 4.2% (1.2%–14%) in 51–60 years (*p* = 0.004); 0.5% (0.2%–1%) to 3.7% (1%–12.5%) in 61–70 years (*p* = 0.038); 0.1% (0%–0.3%) to 3.8% (1.6%–8.6%) in 71–80 years (*p* < 0.001); 0.2% (0.1%–0.4%) to 2.3% (0.6%–8.1%) in 81–90 years (*p* = 0.014); 0.1% (0%–0.6%) to 10% (2.8%–30.1%) over 91 years (*p* = 0.001).

Rhinovirus/enterovirus increased from 34% (30.8%–36.4%) to 41% (35.8%–46.2%) under 2 years (*p* = 0.014); 5.3% (4.2%–6.6%) to 16% (9.1%–25.3%) in 21–30 years (*p* = 0.001); 8.0% (6.6%–9.6%) to 16% (9.4%–25%) in 31–40 years (*p* = 0.015); 1.8% (1.3%–2.6%) to 9.3% (4%–19.9%) in 61–70 years (*p* = 0.004).

RSV increased from 11% (9.5%–13.2%) to 27% (22.9%–32.3%) under 2 years (*p* < 0.001); 6.0% (4.7%–7.7%) to 13% (9.8%–17.8%) in 3–10 years (*p* < 0.001); 0.1% (0%–0.7%) to 2.3% (0.6%–8%) in 11–20 years (*p* = 0.029); 0.4% (0.2%–0.9%) to 6.5% (2.8%–14.3%) in 21–30 years (*p* < 0.001); 1.3% (0.8%–2.1%) to 4.8% (1.9%–11.7%) in 31–40 years (*p* = 0.031); 0.4% (0.2%–0.9%) to 6.2% (2.1%–16.8%) in 51–60 years (*p* = 0.002); 0.2% (0.1%–0.6%) to 5.6% (1.9%–15.1%) in 61–70 years (*p* < 0.001); 0.2% (0.1%–0.5%) to 4.7% (1.8%–11.4%) in 81–90 years (*p* < 0.001).

### 3.5 Age‐Specific Changes in SARS‐CoV‐2 Positivity Rates Before and After Relaxation

We performed an age‐stratified analysis of SARS‐CoV‐2 across various age groups. Of note, the positivity rates of SARS‐CoV‐2 varied among age groups when comparing the pre‐relaxation and post‐relaxation periods (Figure 3A,B and Table S2). SARS‐CoV‐2 decreased from 11% (95% CI: 9.3%–13%) to 5.3% (3.3%–8.2%) under 2 years (*p* = 0.002, chi‐squared test); 18% (16%–21%) to 9.2% (6.3%–13%) in 3–10 years (*p* < 0.001). Conversely, its positivity rate increased from 5.5% (4.5%–6.7%) to 13% (6.4%–24%) in 61–70 years (*p* = 0.032); 3.5% (2.9%–4.2%) to 15% (10%–22%) in 71–80 years (*p* < 0.001); 5.3% (4.5%–6.2%) to 11% (5.6%–19%) in 81–90 years (*p* = 0.048); 5.2% (4.0%–6.8%) to 20% (8.1%–42%) over 91 years (*p* = 0.021). These findings suggest that the spread of SARS‐CoV‐2 infections showed age‐dependent variations after the relaxation of measures.

## 4 Discussion

In this study, we tracked the changes in the prevalence of respiratory pathogens in individuals of different age groups following the regulatory relaxation of restrictions in Japan, prompted by changes in the legal framework for COVID‐19. Our results reveal a significant increase in the positivity rates of respiratory viruses across a wide age range, from pediatric to elderly patients, after the measures were eased. Interestingly, the types of pathogens with increased positivity rates varied between different age groups.

Previous data showed epidemiologic trends in infectious diseases before and during the COVID‐19 pandemic [17]. For instance, Ujiie et al. reported an unusually high prevalence of RSV among children in Tokyo in July 2021 [18]. Similarly, Japan experienced untimely RSV outbreaks in 2021 [19, 20]. Moreover, rhinovirus/enterovirus has been shown to increase during the COVID‐19 pandemic in Japan [21]. In studies from other countries, RSV, parainfluenza 1, and influenza A (H3N2) have surged in children after the relaxation of COVID‐19 restrictions [12, 22, 23, 24]. Our ongoing surveillance found that influenza A and B infections also increased in Japan in winter 2023–2024 (data not shown). While our data support these findings, it also provide further long‐term evidence that shows a significant peak in RSV infections after the relaxation of measures. Rhinovirus/enterovirus also continued to show a gradual increase in positivity rates after the measures were relaxed, with children under 10 years being the most positive.

Regarding age‐stratified data, a previous study showed that children were more likely to be infected with respiratory pathogens than elderly individuals [25]. Interestingly, our data of age‐specific changes revealed variations in the increase or decrease of positivity rates for different respiratory pathogens among various age groups. For example, there was an increase in specific pathogens (adenovirus, pertussis, parainfluenza 2, and parainfluenza 4) predominantly in children under 10 years after the relaxation of measures. Conversely, certain pathogens (human metapneumovirus, rhinovirus/enterovirus, and RSV) increased across a wide age range. Furthermore, SARS‐CoV‐2 showed the different positivity rate among different age groups after the relaxation, with a decrease in children under 10 years but an increase in those over 60 years. Given the limited research on age‐stratified assessments following the country's policy relaxation [26], our study is the first to demonstrate that the behavior of infection spread varies among individual pathogens.

This study has several limitations. These variations are likely influenced by a complex interplay of factors, including social behaviors, vaccination history, seasonality, health literacy, event hosting, international travel, and prior infections [27, 28, 29, 30]. Analyzing these potential confounders by age group is expected to elucidate the underlying causes of changes in pathogen prevalence. In addition, as the results are derived from a single institution, further verification is needed in subsequent studies. Furthermore, data beyond May 8, 2023, are limited, and our data represent only an interim snapshot in time. Therefore, we emphasize the need to evaluate changes in positivity rates from a long‐term perspective.

These data highlight the dynamic changes of circulating respiratory pathogens following regulatory relaxation. This study provides essential epidemiologic data crucial for shaping public health policies and interventions in the post‐COVID‐19 era. Our results emphasize the importance of continuous long‐term surveillance of respiratory infections for a better understanding of infectious disease dynamics and age‐specific trends.

## 5 Conclusion

This study highlights the dynamic landscape of respiratory pathogens after regulatory relaxation following the COVID‐19 pandemic in Japan. The significant increase in positivity rates across different age groups highlights the complex interplay of factors influencing the prevalence of respiratory viruses. These findings serve as a clarion call for adaptive public health strategies, emphasizing the need for tailored interventions to protect different age groups. Long‐term surveillance evidence is critical for effective responses to respiratory infections and informed public health policy‐making in the post‐COVID‐19 era.

## Author Contributions

**Yosuke Hirotsu:** Conceptualization; Data curation; Funding acquisition; Investigation; Visualization; Writing – original draft. **Yuki Nagakubo:** Data curation; Formal analysis; Investigation; Resources. **Makoto Maejima:** Data curation; Formal analysis; Investigation; Resources. **Masahiro Shibusawa:** Data curation; Formal analysis; Investigation; Resources. **Kazuhiro Hosaka:** Data curation; Formal analysis; Investigation; Resources. **Hitomi Sueki:** Formal analysis; Investigation; Resources. **Hitoshi Mochizuki:** Conceptualization; Project administration; Supervision. **Masao Omata:** Conceptualization; Supervision; Writing – review and editing.

## Conflicts of Interest

The authors declare no conflicts of interest.

### Peer Review

The peer review history for this article is available at https://www.webofscience.com/api/gateway/wos/peer‐review/10.1111/irv.13278.

## Supporting information


**Figure S1** Changes in various pathogens by age group before and after relaxation. Scatter plots show the relationship between changes before and after relaxation and their statistical significance. These plots represent other pathogens not shown in Figure 3B. The horizontal axis represents the difference in frequencies (Difference) between after and before relaxation in percentage points. The vertical axis shows the reciprocal of the log_10_ value of the p‐value calculated by the chi‐squared test, indicated as ‐log10(p‐value).

**Figure S2** Changes in various pathogens by age group before and after relaxation. The spider plot represents the positivity rates of various pathogens before (blue line) and after (orange line) the relaxation. The outer boundary represents a frequency of 40%, with each concentric circle indicating a 10% difference.

**Table S1.** Dataset in this study.

**Table S2.** Age class and pathogens

## Data Availability

The dataset supporting the conclusions of this article is included within the article and its additional files.
