# Supplementary figures and images for: Changes in Viral Dynamics Following the Legal Relaxation of COVID‐19 Mitigation Measures in Japan From Children to Adults: A Single Center Study, 2020–2023
Source: Influenza Other Respir Viruses. 2024 Mar 21;18(3):e13278. doi: 10.1111/irv.13278 (PMC10957242; doi:10.1111/irv.13278)

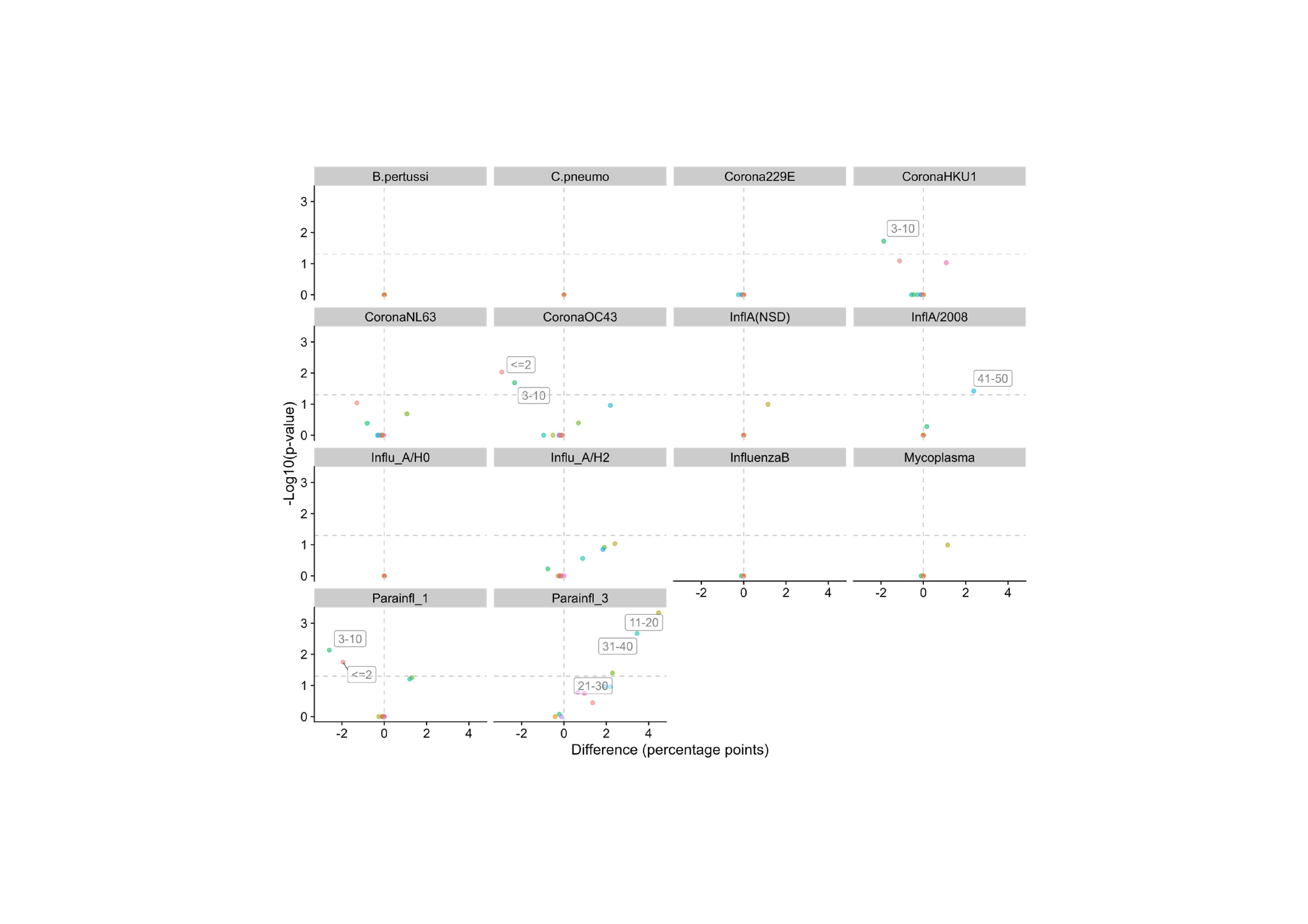

Supplement: Supplementary file 1 — Figure S1 Changes in various pathogens by age group before and after relaxation. Scatter plots show the relationship between changes before and after relaxation and their statistical significance. These plots represent other pathogens not shown in Figure 3B. The horizontal axis represents the difference in frequencies (Difference) between after and before relaxation in percentage points. The vertical axis shows the reciprocal of the log10 value of the p‐value calculated by the chi‐squared test, indicated as ‐log10(p‐value). [file IRV-18-e13278-s002.jpg]

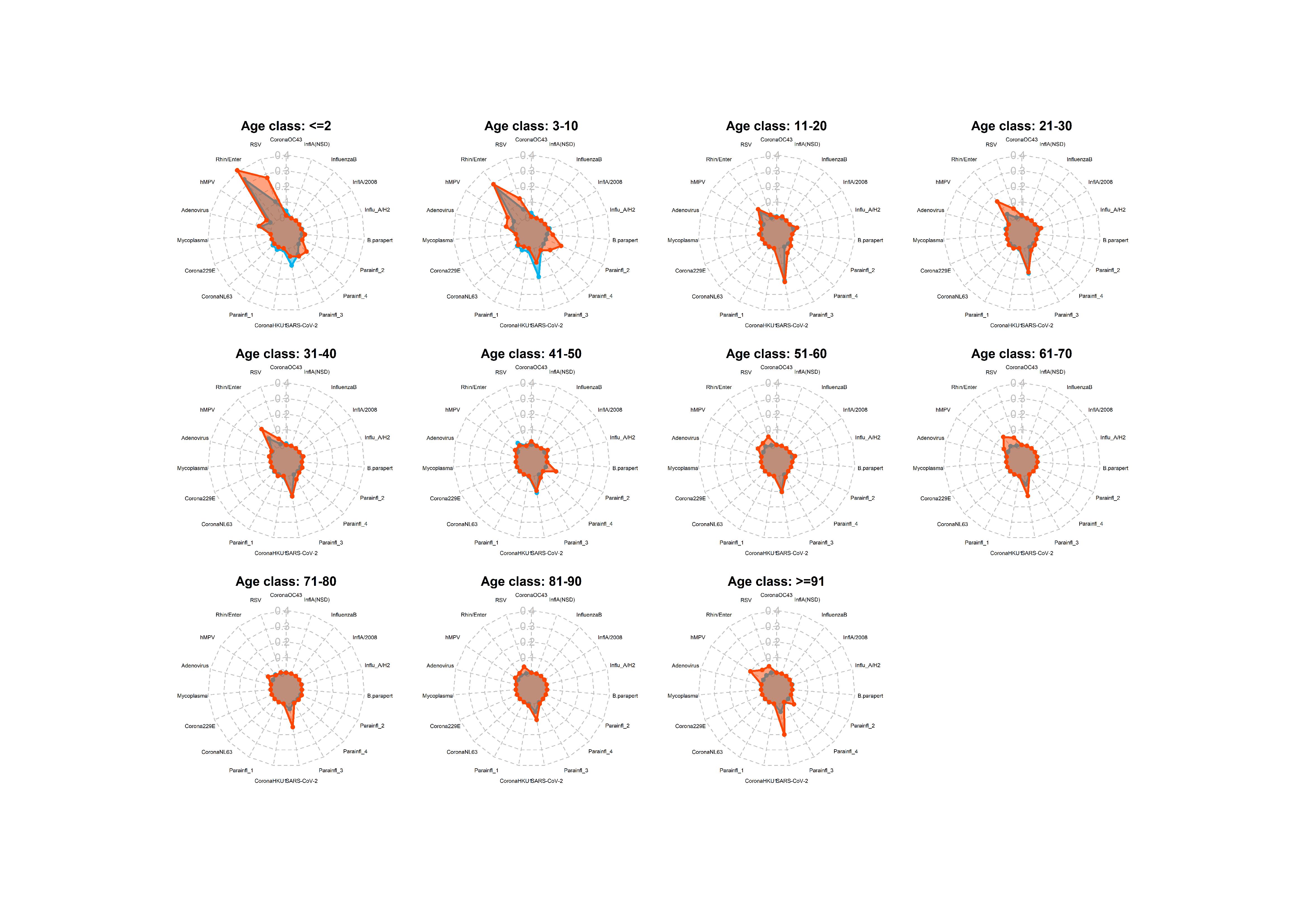

Supplement: Supplementary file 2 — Figure S2 Changes in various pathogens by age group before and after relaxation. The spider plot represents the positivity rates of various pathogens before (blue line) and after (orange line) the relaxation. The outer boundary represents a frequency of 40%, with each concentric circle indicating a 10% difference. [file IRV-18-e13278-s001.jpg]
